# The mediating role of emotional intelligence in the relationship between resilience and workplace violence competence among nursing students

**DOI:** 10.1186/s12912-025-03461-7

**Published:** 2025-07-01

**Authors:** Shaimaa Mohamed Amin, Doaa El Demerdash, Ahmed Abdelwahab Ibrahim El-Sayed, Ahmed Abdellah Othman, Mohamed Hussein Ramadan Atta, Ali Albzia, Mahitab Mohamed Abdelrahman, Haitham Mokhtar Mohamed Abdallah, Ibrahim Alasqah, Shadia Ramadan Morsy Mohamed

**Affiliations:** 1 Community Health Nursing Department, Faculty of Nursing, Damanhour University, Damanhour City, Egypt; 2 Nursing Education Department, faculty of nursing , Damanhour University, Damanhour, Egypt; 3 Faculty of nursing, Rashid University, Egypt; 4 Nursing Administration Department, Faculty of nursing, Alexandria University, Alexandria, Egypt; 5 Nursing Administration Faculty of Nursing -Sohag University, Sohag, Egypt; 6 Faculty of Nursing, Psychiatric and Mental Health Nursing, Alexandria University, Alexandria City, Egypt; 7 Nursing Department College of Applied Medical Sciences - Wadi Aldawasir Campus, Prince Sattam Bin Abdulaziz University, Wadi Addawasir, Saudi Arabia; 8 Nursing Administration and Education Department, College of Nursing, Prince Sattam Bin Abdulaziz University, Al-Kharj, Saudi Arabia; 9 Nursing Administration Department, Faculty of Nursing, Suez Canal University, Ismailia city, Egypt; 10 Faculty of Nursing, Critical Care and Emergency Nursing, Alexandria University, Alexandria City, Egypt; 11 Department of Psychiatric and Mental Health, and Community Health, College of Nursing, Qassim University, Buraydah, 51452 Saudi Arabia; 12 Faculty of Nursing, Nursing Education Department, Alexandria University, Alexandria City, Egypt

**Keywords:** Emotional intelligence, Resilience, Workplace violence, Nursing, Students, Competence, Mediation analysis

## Abstract

**Background:**

Nursing students encounter workplace violence during clinical practice, which poses challenges to their resilience and competence in managing such situations. Emotional intelligence plays a crucial role in how students navigate these challenges, potentially mediating the relationship between resilience and workplace violence competence.

**Aim:**

The primary aim of this study was to evaluate the mediating role of emotional intelligence in the relationship between resilience and the competence to manage workplace violence among nursing students.

**Methods:**

A cross-sectional descriptive design was employed, with 500 undergraduate nursing students from Sohag University participating in the study. Data were collected using three validated scales: the Trait Meta-Mood Scale (TMMS-24) to assess emotional intelligence, the Connor-Davidson Resilience Scale (CD-RISC-10) to measure resilience, and the Management of Workplace Violence Competence Scale (MWVCS) to evaluate competence in managing workplace violence. Data were analyzed using Pearson’s correlation and mediation analysis via JASP software.

**Results:**

Significant positive correlations were found between emotional intelligence and resilience (*r* = 0.42, *p* < 0.001), emotional intelligence and workplace violence competence (*r* = 0.38, *p* < 0.001), and resilience and workplace violence competence (*r* = 0.44, *p* < 0.001). Mediation analysis revealed that emotional intelligence partially mediated the relationship between resilience and workplace violence competence (indirect effect = 0.12, 95% CI [0.08, 0.17]).

**Conclusion:**

The findings highlight the importance of emotional intelligence and resilience in enhancing nursing students’ competence in managing workplace violence. Developing emotional intelligence may be a valuable strategy for improving students’ resilience and readiness to handle challenging clinical environments. Educational programs should consider integrating emotional intelligence training to better equip nursing students for clinical practice.

**Clinical trial number:**

Not applicable.

## Introduction

Workplace violence is a significant issue in healthcare, particularly affecting nursing students who are still developing their professional competencies [1]. Exposure to such violence can undermine their confidence, emotional stability, and overall preparedness for transitioning into full-time practice [2, 3]. Emotional intelligence (EI) and resilience have emerged as critical factors that equip nursing students with the skills to navigate high-stress, confrontational environments more effectively [4].

EI, which involves recognizing, understanding, and managing emotions in oneself and others, plays a vital role in healthcare settings [5]. Studies indicate that nursing students with higher EI are better equipped to manage workplace violence by regulating their emotional responses and understanding others’ emotions [6]. Similarly, resilience enables students to rebound from challenging experiences like workplace violence [7]. While both EI and resilience are crucial, their combined effect on workplace violence competence remains underexplored [8]. This study aims to bridge this gap by examining the mediating role of EI in the relationship between resilience and workplace violence competence among nursing students.

## Background

Resilience, the ability to recover and adapt to adversity, is essential for nursing students facing the challenges of clinical practice and education [6]. In high-pressure healthcare environments, stressors such as heavy workloads and workplace violence can negatively impact their psychological well-being and academic performance [9]. Studies highlight resilience as a protective factor that helps individuals cope and thrive despite these challenges [5, 6]. Among nursing students, resilience is linked to better mental health, academic success, and professional growth [10].

Resilience in nursing students is shaped by individual, social, and environmental factors [11]. Protective elements like social support, mentorship, and coping resources enhance resilience, while unaddressed stressors, including workplace violence, can lead to burnout, anxiety, and reduced clinical performance [12]. Resilience is not solely innate but can be developed through interventions like mindfulness training and stress management programs [13]. However, research remains limited on how resilience interacts with EI to influence nursing students’ ability to manage workplace violence effectively [14].

Lazarus and Folkman’s Stress and Coping Theory (1984) highlighted the interaction between individuals and their environment when facing stressors like workplace violence [15]. This theory involves primary appraisal, where individuals assess threats, and secondary appraisal, where they evaluate coping resources. Resilience and EI play key roles in secondary appraisal, helping individuals manage stress effectively. Resilient individuals adapt to adversity, while those with high EI regulate emotions and navigate social challenges, enhancing their ability to handle workplace violence [16].

EI refers to the ability to perceive, understand, and manage emotions in oneself and others, a skill crucial for effective interpersonal interactions in healthcare settings [1]. For nursing students, EI is particularly important, as it supports compassionate care, effective communication, and conflict resolution. In the context of workplace violence, EI equips nursing students with the tools to de-escalate tense situations, manage their emotional responses, and maintain professionalism under pressure [3].

According to Bar-On’s Emotional Intelligence Theory (1997), EI encompasses a set of emotional and social skills that influence how effectively individuals understand and manage their emotions and relationships [4]. Bar-On’s model is particularly relevant for nursing students who frequently interact with diverse individuals in emotionally charged situations [5]. In this context, EI serves as a mediator that enhances the ability to translate resilience into practical skills for managing workplace violence, such as conflict resolution and de-escalation strategies [1].

The development of EI in nursing education is receiving increasing attention, as it has been linked to better clinical decision-making and patient outcomes [6]. Programs that incorporate reflective practice, simulation-based learning, and emotional regulation strategies have been shown to improve EI among nursing students [7]. However, despite the growing recognition of EI’s importance, its role as a mediator in relationships between other critical traits, such as resilience, and competencies, such as handling workplace violence, is underexplored.

Workplace violence competence refers to the ability to effectively recognize, manage, and mitigate incidents of violence in healthcare settings [24]. For nursing students, this competence is essential, as they are often exposed to aggression from patients, families, or even colleagues during clinical placements [9]. Studies show that workplace violence affects up to 60% of nursing students globally, resulting in psychological distress, reduced confidence, and reluctance to pursue nursing careers [9, 10]. Effective training in workplace violence management, including de-escalation techniques and assertiveness skills, is therefore vital for equipping nursing students with the necessary tools to handle such incidents [11].

Research underscores the significance of both individual and systemic factors in shaping workplace violence competence [28]. While institutional policies and supportive environments play a crucial role, personal attributes such as resilience and EI are pivotal in determining an individual’s ability to effectively navigate violent situations [29]. Nursing students with greater resilience demonstrate higher composure and resourcefulness in violent encounters, whereas those with enhanced EI exhibit superior emotional regulation and communication skills [30, 31]. Despite these insights, gaps persist in understanding the interplay between these factors in developing workplace violence competence, particularly during the early stages of nursing education.

Furthermore, this study incorporates Bandura’s Social Cognitive Theory (1986), which highlights the role of self-efficacy in shaping behavior [32]. Workplace violence competence is closely linked to nursing students’ confidence in their ability to manage violent situations. Resilience and EI contribute to this self-efficacy by fostering a sense of control and competence in high-stress scenarios [33, 34]. Bandura’s framework posits that individuals acquire skills through observation, modeling, and experience, suggesting that interventions aimed at enhancing resilience and EI could significantly strengthen workplace violence competence by promoting adaptive behavioral responses and effective emotional regulation strategies [35].

### Study hypotheses

#### H_1_

Resilience is positively associated with workplace violence competence among nursing students.

#### H_2_

EI is positively associated with workplace violence competence among nursing students.

#### H_3_

EI mediates the relationship between resilience and workplace violence competence among nursing students.

## Methods

### Study design and setting

This study employed a cross-sectional descriptive research design, following the Strengthening the Reporting of Observational Studies in Epidemiology (STROBE) guidelines. The research was conducted at the Faculty of Nursing, Sohag University, located in Sohag Governorate, Egypt.

### Sample size and study participants

The target group for this study comprised undergraduate nursing students. To be eligible for participation, students needed to meet the following criteria: currently enrolled in the Faculty of Nursing for the 2024–2025 academic year, have clinical placement experience with exposure to workplace environments, and express a willingness to participate in the study. Students without clinical experience or those unwilling to participate were excluded.

In the Faculty of Nursing where this study was conducted, undergraduate students begin their clinical placements in the second year of their program. Clinical training continues throughout the remainder of their studies, integrating both hospital and community-based settings. The duration of clinical experience varies by year, with students engaging in approximately 120 h of supervised clinical practice per semester. By the time of participation in this study, students had completed at least 100 h of clinical exposure, ensuring their familiarity with workplace environments and potential experiences related to workplace violence. In addition to clinical placements, the undergraduate nursing curriculum includes elements of emotional intelligence and resilience integrated within several core courses, such as communication skills, professional development, and community health nursing. These courses expose students to concepts related to emotional regulation, empathy, stress management, and interpersonal communication. However, the program does not currently offer a dedicated course specifically focused on emotional intelligence or resilience as standalone subjects.

**Fig. 1:**
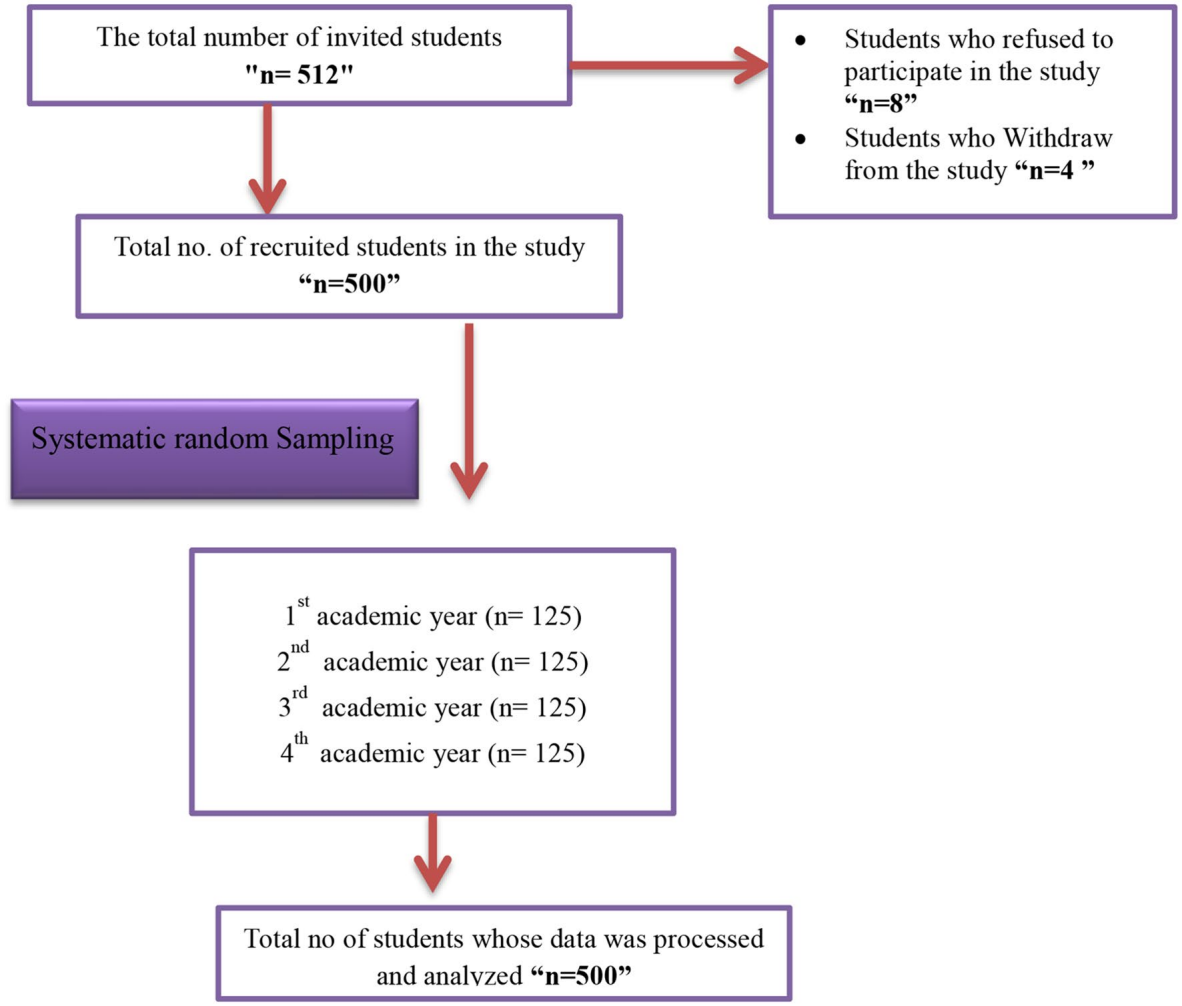
Flow chart of participants’ recruitment

Researchers employed the G* Power version 3.1.9.7 software program [12]to calculate the sample size. The specified parameters for the calculation included an estimated effect size of 0.15 based on previous study on emotional intelligence among nursing students [13], α error probability of 0.05, and Power (1-β error probability) of 0.90. The program recommended a minimum total sample size of 480. However, the researchers decided to recruit 512 students to compensate for possible non-response. The final sample included 500 participants who consented to complete the questionnaire. To achieve a balanced representation across all academic years, an equal allocation method was used, with 125 students selected from each year. Systematic random sampling was employed by selecting every 20th student from a predefined list or population, ensuring a randomized and representative sample across the entire cohort as shown in Fig. 1.

### Measurements

#### The demographic form

The form gathers socio-demographic information, including age, gender, academic year, place of residence, family structure, income level, marital status, parents’ educational background, and any previous exposure to workplace violence during clinical practice.

### Emotional intelligence scale

The Trait Meta-Mood Scale (TMMS-24), originally developed by Salovey et al. in 1995 [14], is designed to assess trait emotional intelligence through its three dimensions: emotional perception, emotional comprehension, and emotional regulation. Each dimension comprises 8 items, rated on a five-point Likert scale from strongly disagree [1] to strongly agree [5]. The total score ranges from 24 to 120, with higher scores indicating greater trait emotional intelligence. Espinoza et al. [15] validated the TMMS-24 among nursing students, demonstrating robust reliability with Cronbach’s alpha values above 0.85 for all dimensions. The Arabic version of the TMMS-24 demonstrated strong psychometric properties. Exploratory Factor Analysis (EFA) revealed a clear three-factor structure, with factor loadings ranging from 0.65 to 0.92 after varimax rotation, accounting for 75.2% of the total variance. Sampling adequacy was excellent (KMO = 0.940), and Bartlett’s test of sphericity was highly significant (*p* ≤ 0.001), confirming the suitability of the data for factor analysis. Confirmatory Factor Analysis (CFA) using Maximum Likelihood Estimation supported the three-factor model, showing good fit indices (χ² = 458.23, df = 227, *p* < 0.001; χ²/df = 2.02; CFI = 0.954; TLI = 0.947; RMSEA = 0.049; SRMR = 0.041). Reliability testing indicated high internal consistency (Cronbach’s alpha = 0.89 for the total scale) and excellent stability over a two-week interval (Intraclass Correlation Coefficient = 0.91), confirming that the Arabic TMMS-24 is a valid and reliable tool for assessing emotional intelligence in nursing students.

### Management of workplace violence competence scale (MWVCS)

The Management of Workplace Violence Competence Scale (MWVCS), developed by Lu et al. (2021) [9], assesses nursing students’ ability to manage workplace violence during clinical practicum. It consists of 40 items across seven dimensions: After-the-Event Recovery (11 items), Nurse-Patient Interaction (6 items), Response to Violence (8 items), Violence Cognition (5 items), Utilization of Protective Facilities (4 items), Knowledge Renewal (3 items), and Risk Assessment (3 items). Items are rated on a five-point Likert scale from strongly disagree [1] to strongly agree [5], with higher scores indicating greater competence. In this study, the scale’s reliability was further supported by a Cronbach’s alpha of 0.922. Post-translation validity into Arabic was confirmed by EFA, showing factor loadings between 0.65 and 0.97 after varimax rotation, surpassing the 0.40 threshold and accounting for 75.23% of total variance. The Kaiser-Meyer-Olkin (KMO) measure was strong at 0.950, and Bartlett’s test of sphericity was highly significant (*p* ≤ 0.001), validating the data for factor analysis and retaining all items on the scale. To further establish the structural validity of the Arabic version, Confirmatory Factor Analysis (CFA) was conducted using Maximum Likelihood Estimation in AMOS 28. The results confirmed the seven-factor structure, demonstrating an acceptable model fit (χ² = 612.45, df = 335, *p* < 0.001; χ²/df = 1.83; CFI = 0.953; TLI = 0.946; RMSEA = 0.048; SRMR = 0.039). These findings indicate that the Arabic MWVCS retains the intended factor structure of the original scale.

To assess the stability over time, test-retest reliability was conducted over a two-week interval, yielding an intraclass correlation coefficient (ICC) of 0.91, reflecting excellent temporal stability. These psychometric evaluations confirm that the Arabic version of the MWVCS is a valid and reliable instrument for assessing nursing students’ competence in managing workplace violence.

### Connor–Davidson resilience scale (10- item CD-RISC)

The Connor-Davidson Resilience Scale (CD-RISC) was originally developed by Connor and Davidson in 2003 [16] to measure resilience, or the ability to adapt and recover from adversity. Campbell-Sills and Stein (2007) [17] later refined and validated the CD-RISC, presenting a 10-item version. Each item is rated on a five-point Likert scale, from “Not true at all” [1] to “True nearly all the time” [5], with higher scores indicating greater resilience. In this study, the Arabic-translated CD-RISC-10 showed robust reliability, with a Cronbach’s alpha of 0.91. Exploratory factor analysis (EFA) confirmed its validity, with factor loadings ranging from 0.55 to 0.88, improving to 0.70 to 0.93 after varimax rotation, and explaining 77.45% of the variance. The Kaiser-Meyer-Olkin (KMO) measure was excellent at 0.930, and Bartlett’s test of sphericity was highly significant (*p* ≤ 0.001), validating the suitability of the data for factor analysis. All items were retained on the scale. To further confirm the factorial structure, Confirmatory Factor Analysis (CFA) was conducted using Maximum Likelihood Estimation in AMOS 28. The results supported the one-factor structure, yielding an adequate model fit (χ² = 64.21, df = 34, *p* < 0.001; χ²/df = 1.89; CFI = 0.961; TLI = 0.952; RMSEA = 0.046; SRMR = 0.037), reinforcing the structural validity of the Arabic CD-RISC-10. To assess the scale’s stability over time, test-retest reliability was evaluated over a two-week interval, yielding an intraclass correlation coefficient (ICC) of 0.89, demonstrating excellent temporal stability.

### Study procedures

Prior to data collection, all research instruments were subjected to a rigorous adaptation process to ensure linguistic and cultural suitability for Arabic-speaking participants. The original tools were translated into Arabic by bilingual experts fluent in both English and Arabic. To ensure linguistic equivalence, a back-translation method was employed, in which independent translators re-translated the Arabic versions into English.

An expert panel consisting of five nursing and psychology faculty members assessed the face and content validity of the translated instruments. The experts evaluated the items for clarity, relevance, and cultural appropriateness within the context of Arabic-speaking nursing students. Minor modifications were made based on their feedback. Subsequently, a pilot study was conducted with 30 undergraduate nursing students to evaluate the clarity, comprehensibility, and reliability of the translated instruments. These participants were excluded from the main study. The pilot results indicated satisfactory item clarity and internal consistency, and no further revisions were necessary.

### Data collection

Data collection for this study took place from September to November 2024, following the receipt of necessary permissions. Before starting, the researchers explained the study’s objectives to each student, highlighting the voluntary nature of participation. Written informed consent was obtained from each participant as a prerequisite for involvement. To encourage trust, the researchers assured participants of the confidentiality of their responses. Questionnaires were distributed in quiet locations, such as empty lecture halls and libraries, between 9 am and 2 pm from Saturday through Thursday. Most participants completed the questionnaire in approximately 10 to 15 min.

### Data analysis

Researcher analysis the collected data by SPSS 26.0 (IBM Inc., Chicago, IL, USA) to examine the survey responses from the 500 nursing students. Frequencies (number and percentage) and mean ± standard deviations (S.D.) were used to summarize general characteristics of participants and for descriptive statistics. Construct validity was assessed using confirmatory factor analysis (CFA) and (EFA) with maximum likelihood estimation using AMOS v. 21. Cronbach’s alpha and McDonald’s omega coefficient were used to assess the reliability of the survey. The correlations between emotional intelligence, management of workplace violence competence and resilience were evaluated by Pearson’s correlation analysis. JASP 0.14.1.0 was used for testing the mediating role of emotional intelligence between resilience and management of workplace violence competence.

## Results

The characteristics of participants are presented in Table 1 and 50.4% of the participants were less than 20 years, 56.6% were from rural areas, 62.4% of them had enough income and 95.6% were single. Furthermore, 35.8% of their mothers had secondary education, 35.6% of their fathers had university and more educational level and 75% didn’t exposure to violence in the clinical setting.

**Table 1:** Personal characteristics among participants (*n* = 500)

Personal data	Categories	No	%
**Age**	less than 20 years	252	50.4
	20 to less than 22	147	29.4
	22 to less than 24	97	19.4
	more than 24	4	0.9
**Residence**	Rural	283	56.6
	Urban	217	43.4
**Monthly Income**	Not enough	76	15.2
	Enough	312	62.4
	enough and save	112	22.4
**Marital status**	single	478	95.6
	married	22	4.4
**Mother education**	illiterate	77	15.4
	basic education	73	14.6
	secondary	179	35.8
	university and more	171	34.2
**Father education**	illiterate	44	8.8
	basic education	107	21.4
	secondary	171	34.2
	university and more	178	35.6
**Previous exposure to violence in the clinical setting**	Yes	125	25.0
	No	375	75.0

The average mean score of the participants emotional intelligence scale was 3.45 ± 0.466 with the subscales were 3.77 ± 0.461 for cognitive identity, 4.39 ± 0.704 for emotional identity, 3.80 ± 0.493 for behavioral identity. Furthermore, the average mean score of the participants management of workplace violence competence scale was 3.41 ± 0.596 with the subscales; after-the-event recovery was 3.25 ± 1.071, nurse-patient interaction was 3.2792 ± 0.91453, response to violence was 3.74 ± 0.929, violence cognition was 3.28 ± 0.553, utilization of protective facilities was 3.1943 ± 0.48258, knowledge renewal was 3.34 ± 0.843 and 3.65 ± 0.943 for risk assessment. In addition, the average mean score of the participants’ resilience was 3.62 ± 1.607. See Table 2.

**Table 2:** Descriptive analysis between study variables (*n* = 500)

Variables	*N*	Minimum	Maximum	Mean ± SD	Average mean ± SD
Cognitive Identity	8	8	40	30.49 ± 1.837	3.77 ± 0.4618
Emotional Identity	8	10	40	29.58 ± 2.847	4.39 ± 0.7043
Behavioral Identity	8	9	40	33.92 ± 1.874	3.80 ± 0.493
**Emotional intelligence**	24	40.00	120.00	87.488 ± 16.07	3.45 ± 0.466
After-the-Event Recovery	11	15	55	28 ± 2.35622	3.25 ± 1.071
Nurse-Patient Interaction	6	9	30	19 ± 3.48718	3.27 ± 0.914
Response to Violence	8	10	40	27.39 ± 0.946	3.74 ± 0.929
Violence Cognition	5	5	25	19.36 ± 4.653	3.28 ± 0.553
Utilization of Protective Facilities	4	7	20	14.04 ± 2.082	3.19 ± 0.482
Knowledge Renewal	3	6	15	8.94 ± 4.643	3.346 ± 0.843
Risk Assessment	3	5	15	10.38 ± 2.646	3.65 ± 0.943
**Management of Workplace Violence Competence**	40	81.00	199.00	149.03 ± 29.235	3.41 ± 0.596
**Resilience**	10	10.00	50.00	39.47 ± 7.162	3.625 ± 1.607

SD = Standard deviation

**Fig. 2:**
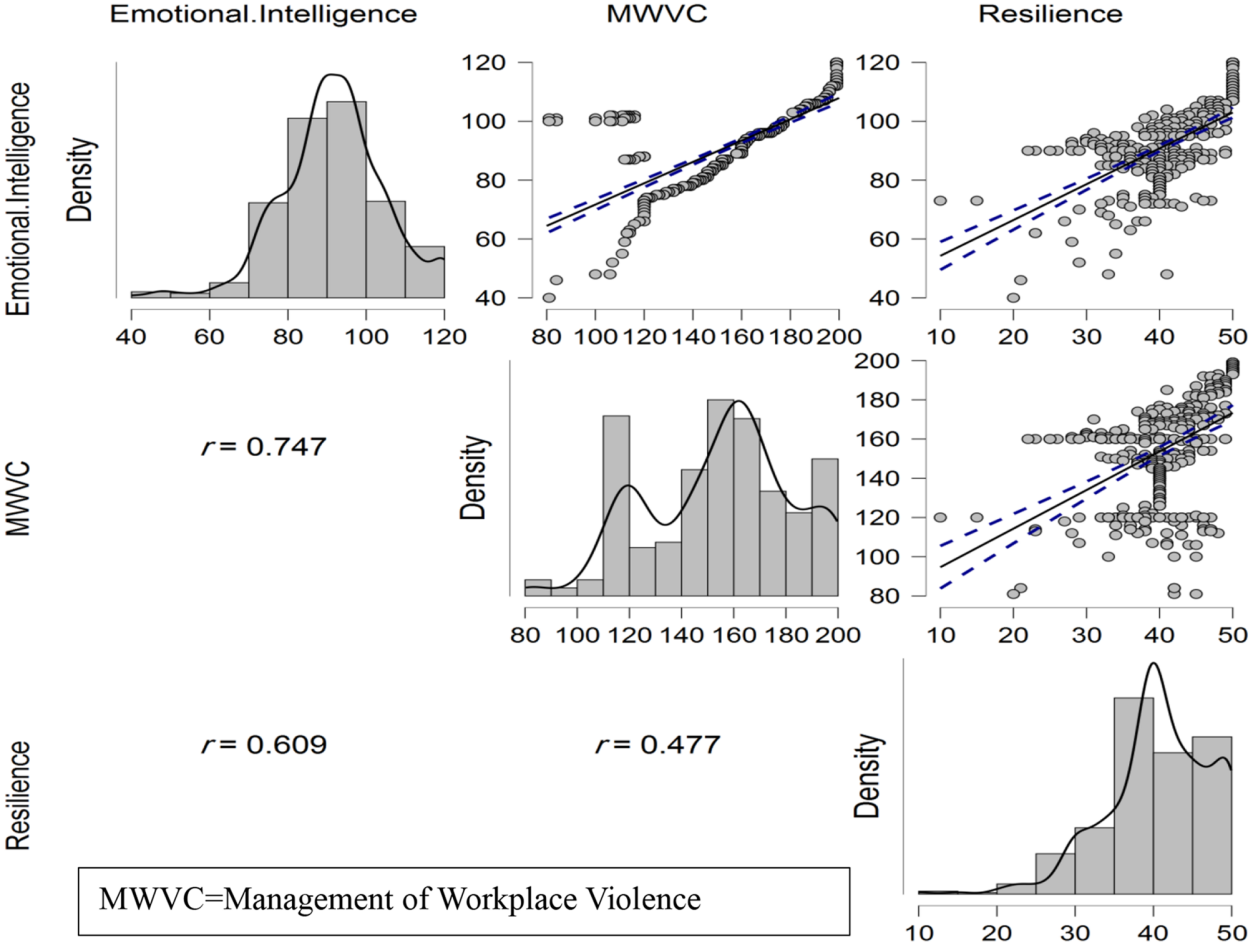
Density and correlation analysis between study variables (*n* = 500)

There was statistical significant positive correlation between emotional intelligence and management of workplace violence competence *r* = 0.747^**^, *p* < 0.001^**^ and between emotional intelligence and resilience at *r* = 0.609^**^, *p* < 0.001^**^, also, there was positive correlation between management of workplace violence competence and resilience at *r* = 0.477^**^, *p* < 0.001^**^. See Table 3; Fig. 2.

**Table 3:** Correlation analysis between study variables (*n* = 500)

Variables		Emotional intelligence	Management of workplace violence competence	Resilience
Emotional intelligence	r	1	0.747^**^	0.609^**^
	P	-	< 0.001^**^	< 0.001^**^
Management of Workplace Violence Competence	r		1	0.477^**^
	P		-	< 0.001^**^
Resilience	r			1
	P			-

**Correlation is highly significant at the 0.01 level (2-tailed)

**Fig. 3:**
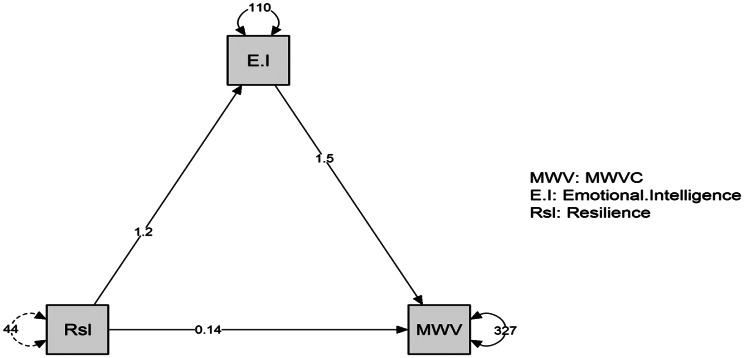
Mediating effect of emotional intelligence between resilience and management of workplace violence competence (*n* = 500)

There was a statistically significant direct effect of resilience on emotional intelligence at B = 0.141, t = 6.723, *p* = 0.021^*^, on management of workplace violence competence at B = 1.542, t = 18.193, *p* < 0.001 and emotional intelligence on management of workplace violence competence at B = 1.542, t = 18.193, *p* < 0.001. Furthermore, there was a statistically significant indirect effect of resilience on management of workplace violence competence when emotional intelligence act as a mediator variables at B = 1.626, t = 11.884, *p* < 0.001. See Table 4; Fig. 3.

**Table 4:** Path model for the effect of emotional intelligence between resilience and management of workplace violence competence (*n* = 500)

Direct effect	(B)	CI 95%	t	*p*
Resilience → Emotional intelligence	1.218	(1.067–1.370)	15.826	< 0.001^**^
Resilience → Management of Workplace Violence Competence	0.141	(0.036-0.224)	6.723	0.021^*^
Emotional intelligence → Management of Workplace Violence Competence	1.542	(1.411–1.673)	18.193	< 0.001^**^
**Indirect effect**				
Resilience → Emotional intelligence → Management of Workplace Violence Competence	1.626	(1.525 --2.128)	11.884	< 0.001^**^
**Total effect**				
Resilience → Management of Workplace Violence Competence	1.768	(1.624–2.312)	11.206	< 0.001^**^

## Discussion

Workplace violence remains a pressing issue in healthcare, particularly in environments where nursing students undergo clinical training. These trainees, who are still developing their professional and emotional skills, face unique challenges in navigating such high-stress scenarios [18]. As incidents of workplace violence can significantly affect their psychological well-being, academic performance, and career readiness, understanding the factors that equip nursing students to handle such situations is critical [19]. The findings of this study underscore the significant interplay between resilience, emotional intelligence (EI), and workplace violence competence, while providing insights into how these attributes collectively prepare nursing students for professional challenges. These relationships provide crucial insights for nursing education and the preparation of students to manage the complexities of clinical practice.

The results demonstrate that emotional intelligence (EI) mediates the relationship between resilience and workplace violence competence, aligning with Bar-On’s Emotional Intelligence Theory, which highlights EI’s role in stress and interpersonal management [4]. EI enhances nursing students’ ability to navigate high-stress environments, particularly in aggressive situations, reinforcing its importance in professionalism by ensuring composure, empathy, and ethical decision-making [20]. Consistent with Hurley et al. (2020) [21], the strong correlation between EI and workplace violence competence emphasizes its role in facilitating empathy, communication, and de-escalation strategies, allowing nursing students to manage adversarial situations professionally [22]. This study confirms that resilience alone is insufficient for violence management competence and that EI strengthens resilience by enhancing emotional regulation, interpersonal understanding, and self-awareness, transforming it into an actionable professional skill [23].

Stress and Coping Theory [24] explains how individuals appraise and respond to stressors. In workplace violence scenarios, resilience influences initial cognitive appraisal, shaping whether aggression is perceived as a manageable challenge or an overwhelming threat. However, EI determines the effectiveness of coping responses, guiding students toward strategic emotional regulation and conflict resolution instead of reactive behaviors [18]. Problem-focused coping, such as assertive communication and conflict de-escalation, is optimized when EI enables students to assess social cues and regulate emotional responses. Emotion-focused coping, including self-reflection and emotional regulation, helps students process workplace violence while maintaining professional integrity [25].

Additionally, Bandura’s (1986) [26] Social Cognitive Theory highlights self-efficacy, which is strengthened through both resilience and EI, fostering confidence in handling workplace aggression professionally. While resilience provides endurance, EI translates it into professional behaviors, such as maintaining composure, ethical decision-making, and effective de-escalation techniques. The interconnection between EI, resilience, and workplace violence competence underscores EI’s essential role in preparing nursing students for real-world clinical challenges [27]. Moreover, by reinforcing the direct link between EI, resilience, and workplace violence competence, these theories clarify how EI enhances aggression management while also shaping professional identity. The ability to regulate emotions, communicate professionally, and uphold ethical standards in high-stress situations is fundamental to nursing professionalism. Integrating EI training into nursing education is crucial to ensuring that students develop the emotional and psychological competencies needed for effective and ethical practice in clinical settings [28].

Resilience was found to directly influence workplace violence competence, affirming its role as a protective factor in high-pressure environments. This aligns with Diffley and Duddle’s (2022) work, which highlights resilience as a key attribute enabling students to recover from and adapt to workplace challenges [29]. Notably, resilience supports nursing students in maintaining composure and resourcefulness during violent encounters, critical for professional success [23, 30]. Furthermore, these findings resonate with Aryuwat et al.’s (2023) integrative review, which confirms the protective nature of resilience against workplace stressors in clinical settings [31]. However, the indirect effects mediated by EI reveal a deeper interconnection. Resilience, foundational, benefits from an additional layer of emotional and social intelligence to translate into workplace violence competence [32]. Ang and Lau (2024) similarly emphasize that resilience coupled with EI creates a synergistic effect, amplifying coping strategies and professional capabilities [33]. This study’s findings highlight the necessity of interventions targeting both resilience-building and EI enhancement to optimize outcomes. Such a multi-dimensional approach aligns with Bandura’s Social Cognitive Theory (1986), which suggests that self-efficacy, strengthened by resilience and EI, enhances individuals’ ability to navigate complex situations confidently [26].

The multifaceted nature of workplace violence competence, encompassing dimensions such as after-the-event recovery, nurse-patient interaction, and risk assessment, illustrates the complexity of managing violence in healthcare [22]. This study reveals that EI contributes significantly to these dimensions, particularly in emotionally charged and communicative aspects. For instance, Mora et al. (2024) demonstrates how EI enhances professionalism and empathy during violent encounters, validating the present findings [7]. EI’s role extends to fostering effective decision-making and de-escalation strategies, critical in ensuring patient and staff safety during confrontational scenarios. Resilience complements these competencies by fostering persistence and adaptability [34]. Nursing students with higher resilience scores displayed superior cognitive and behavioral responses to workplace violence, paralleling Aryuwat et al.’s (2023) findings [31]. Importantly, the interplay of resilience and EI enhances these responses further, underscoring the critical integration of emotional and psychological resources for effective violence management. This dual competency approach not only improves immediate responses but also builds long-term professional resilience, equipping students to handle future challenges [29, 35].

The findings present actionable implications for nursing education. They underscore the necessity of curricula that integrate resilience training with EI development. While traditional nursing education focuses on technical skills, this study emphasizes the importance of psychological and emotional competencies. Such integration addresses the gaps in preparing students for environments where workplace violence is prevalent. Simulation-based learning, reflective practice, and emotional regulation workshops emerge as promising strategies [11, 36]. For example, simulated workplace violence scenarios allow students to apply resilience and EI skills in controlled settings. These interventions have demonstrated success in enhancing both psychological preparedness and practical competence [37]. Additionally, reflective practices enable students to process and learn from their experiences, fostering emotional intelligence and resilience. Evidence suggests that such experiential learning fosters deeper emotional engagement, leading to lasting behavioral changes [1, 6].

The development of emotional intelligence (EI) and resilience plays a critical role in enhancing nursing students’ competence in managing workplace violence [28]. This study emphasizes the need for individual-level interventions that focus on strengthening emotional regulation, adaptive coping strategies, and interpersonal skills. Rather than broad institutional interventions, targeted educational approaches such as simulation-based training, guided self-reflection, and de-escalation skill-building are more directly related to improving students’ ability to navigate aggressive or violent clinical situations with professionalism and confidence [6].

While institutional policies such as zero-tolerance approaches to workplace violence and reporting systems contribute to a supportive learning environment [19], the primary determinant of workplace violence competence is the integration of EI and resilience-building strategies within nursing education [30]. By equipping students with the ability to regulate emotions, assess social cues, and engage in effective conflict resolution, EI enhances resilience and transforms it into a practical skill set for handling workplace aggression [38].

This study underscores the necessity of incorporating evidence-based EI and resilience training into nursing curricula to prepare students for the psychological and professional demands of clinical practice. Future research should explore the effectiveness of specific EI-enhancing interventions and identify barriers to implementation within nursing education, ensuring that students develop the emotional and psychological competencies required for managing workplace challenges effectively [6].

The findings must be interpreted within the context of certain limitations. Cultural and organizational factors in healthcare settings may influence the prevalence and management of workplace violence [39]. This study’s participants, drawn from a specific geographic and educational context, may not represent the diversity of nursing experiences globally. For example, while resilience and EI are universally beneficial, their application may vary depending on regional differences in workplace culture and healthcare policies. Future research should examine these relationships across varied populations to validate and extend these findings [40, 41].

Further investigations should expand on these findings by clarifying the relationship between emotional intelligence (EI), resilience, and workplace violence competence and identifying effective interventions to enhance nursing students’ safety and professional preparedness. Additionally, studies should examine barriers to implementing resilience and EI training, such as resource limitations, resistance to curriculum changes, and varying levels of acceptance among students and faculty [27, 31, 39]. This study highlights the interdependent roles of EI and resilience in shaping nursing students’ competence in handling workplace violence. The findings reinforce the need for concise, targeted educational strategies that integrate EI and resilience training, ensuring students are well-prepared for the challenges of clinical practice. Strengthening these attributes within nursing curricula will enhance students’ ability to navigate workplace aggression professionally, fostering both personal and professional growth.

## Conclusion

This study underscores the mediating role of emotional intelligence in the relationship between resilience and workplace violence competence among nursing students. Higher resilience enhances emotional intelligence, which, in turn, strengthens students’ ability to manage workplace violence. These findings highlight the importance of integrating emotional intelligence training into nursing curricula to better prepare students for clinical challenges. Strengthening these competencies can contribute to a safer and more supportive healthcare environment.

### Implications

The findings of this study have important implications for nursing research, highlighting the need for further exploration of the relationship between emotional intelligence, resilience, and workplace violence competence. Future research could focus on developing targeted interventions to enhance emotional intelligence among nursing students, thereby strengthening their coping mechanisms and preparedness for clinical challenges. Additionally, studies could examine the long-term effects of emotional intelligence training on workplace violence management as students transition into professional practice. These insights could refine existing nursing education frameworks and support the development of more effective, evidence-based training programs.

In the context of nursing education, this study underscores the importance of integrating emotional intelligence and resilience-building strategies into curricula. By incorporating training in emotional intelligence, stress management, and conflict resolution, nursing programs can better prepare students for the emotional demands of clinical practice. In nursing practice, fostering these competencies can enhance nurses’ self-efficacy, equipping them to manage workplace violence and other stressors more effectively. Ultimately, these improvements can contribute to a healthier work environment, better patient care outcomes, and a more supportive and resilient healthcare workforce.

### Strengths and limitations

One of the key strengths of this study is the large, representative sample size of 500 nursing students, which enhances the generalizability of the findings. The use of systematic random sampling ensures that the sample is diverse and reflective of the entire cohort across all academic years. The robust psychometric properties of the instruments used, such as the Trait Meta-Mood Scale (TMMS-24), the Management of Workplace Violence Competence Scale (MWVCS), and the Connor-Davidson Resilience Scale (CD-RISC), contribute to the study’s reliability and validity. Additionally, the study adheres to rigorous ethical standards, ensuring the protection of participants’ rights and confidentiality, and the use of both exploratory and confirmatory factor analysis strengthens the validity of the translated tools in the Arabic context. A notable limitation of this study is its cross-sectional design, which restricts the ability to infer causal relationships between emotional intelligence, resilience, and workplace violence competence. As data was collected at a single point in time, it limits understanding of the long-term effects or changes in these variables. Additionally, the study’s reliance on self-report questionnaires may introduce response bias, as participants might have a tendency to answer in socially desirable ways. The sample was also drawn from a single university in Egypt, which may limit the external validity of the findings to other regions or educational settings. Furthermore, while the study addresses key emotional and competence factors, it does not consider other potential confounders or variables that could influence the outcomes, such as prior personal experiences with workplace violence.

## Data Availability

The datasets generated and analyzed during the current study are notpublicly available due to confidentiality agreements but are available uponreasonable request from the corresponding author.

## References

[1] Ruiz-Ortega AMS-Á, Berrios-Martos MP. Psychological well-being and emotional intelligence in undergraduate nursing students as predictors of academic success. Nurse Educ Today. 2024;1:143. 10.1016/j.nedt.2024.106406 39288607

[2] Napolitano FCM, Pagnucci N, Zanini M, Catania G, Aleo G, Gomes L, Sasso L, Bagnasco A. The effectiveness of learning strategies for the development of emotional intelligence in undergraduate nursing students: A systematic review protocol. Nurse Educ Pract. 2023;1:72103797. 10.1016/j.nepr.2023.103797 37832374

[3] Jawabreh N. The relationship between the emotional intelligence and clinical decision making among nursing students. SAGE Open Nurs 2024:10:23779608241272459. 10.1177/23779608241272459 PMC11307361 39119200

[4] Bar-On RBJ, Kirkcaldy BD, Thome EP. Emotional expression and implications for occupational stress; an application of the emotional quotient inventory (EQ-i). Pers Indiv Differ. 2000;1(6):1107–18.

[5] Dawda DHS. Assessing emotional intelligence: reliability and validity of the Bar-On emotional quotient inventory (EQ-i) in university students. Pers Indiv Differ. 2000;1(4):797–812.

[6] Rodríguez-Leal LG-HR, Silva LI, Rodríguez-Gallego I, Saldaña MR, Montesinos JV. Influence of clinical internship on emotional intelligence as perceived by nursing students: A longitudinal study. Educación Médica 2024; 1;25(5):100936.

[7] Mora MSÁB, Cabodevilla AA, Vázquez-Calatayud M. Emotional intelligence of nurses in intensive care units: A systematic review. Intensive Crit Care Nurs. 2024;1:84. 10.1016/j.iccn.2024.103724 38824712

[8] Bsharat F. Relationship between emotional intelligence and self-esteem among nursing students. SAGE Open Nurs 2024;10:23779608241252248. 10.1177/23779608241252248 PMC11062226 38693934

[9] Lu D, Jeong SYS, Zhu L. Development and validation of a management of workplace violence competence scale for nursing practicum students. Asian Nurs Res. 2021;15(1):23–9. 10.1016/j.anr.2020.10.005 33253928

[10] Wang MCT, Guan H, Yang Y, Da C, Pan Q. Competence in managing workplace violence among nursing interns: Application of latent class analysis. Nurse education in practice 2023; 1;:73:103850. 10.1016/j.nepr.2023.103850 37995448

[11] M. AJ. Enhancing nursing students’ competency skills with a workplace violence nursing simulation: translating knowledge into practice. SAGE Open Nurs. 2019;5:2377960819843696. 33415232 10.1177/2377960819843696 PMC7774437

[12] Faul F, et al. G* power 3: A flexible statistical power analysis program for the social, behavioral, and biomedical sciences. Behav Res Methods. 2007;39(2):175–91. 17695343 10.3758/bf03193146

[13] El-Gazar HE, Elgohari H, Loutfy A, Shawer M, El-Monshed AH, Abou Zeid MAG, et al. Does internet addiction affect the level of emotional intelligence among nursing students? A cross-sectional study. BMC Nurs. 2024;23(1):555. 39135017 10.1186/s12912-024-02191-6 PMC11321225

[14] Salovey PMJ, Goldman S, Turvey C, Palfai T. In: James Pennebaker W, editor. Emotional attention, clarity, and repair: exploring emotional intelligence using the trait meta-mood scale. Washington DC: APA Science Volume Series; 1995. pp. 125–54.

[15] Espinoza-Venegas MS-AO, Ramírez-Elizondo N, Sáez-Carrillo K. A validation of the construct and reliability of an emotional intelligence scale applied to nursing students. Rev Latinoam Enferm. 2015;23(1):139–47. 10.1590/0104-1169.3498.2535 PMC4376042 25806642

[16] Connor KM, Davidson JRT. Development of a new resilience scale: the Connor-Davidson resilience scale (CD-RISC). Volume 18. Depression and Anxiety; 2003. pp. 76–82. 2. 10.1002/da.10113 12964174

[17] Campbell-Sills L, Stein MB. Psychometric analysis and refinement of the connor–davidson resilience scale (CD‐RISC): validation of a 10‐item measure of resilience. J Trauma Stress: Official Publication Int Soc Trauma Stress Stud. 2007;20(6):1019–28. 10.1002/jts.20271 18157881

[18] Al Kadi SS, Beydoun AR, Ali AA. Nurses’ emotional intelligence, behavior and the mediating role of job stress in Lebanon. BAU J - Soc Cult Hum Behav. 2023;4(2).

[19] Alenezi A, Barr L. The impact of resilience on workplace violence experienced by mental health nurses: A Cross-Sectional survey. J Nurs Adm Manag. 2024;2024:1–10. 10.1155/2024/4449445 PMC11918818 40224751

[20] Shaban M, Ezzelregal Abdelgawad M, Mohamed Elsayed S, Mohamed Abdallah HM. The mediating role of emotional intelligence in the relationship between technostress and burnout prevention among critical care nurses a structural equation modelling approach. BMC Nurs. 2025;24(1):255. 40050866 10.1186/s12912-025-02852-0 PMC11887162

[21] Hurley JHM, Kozlowski D, Gadd M, Van Vorst S. Emotional intelligence as a mechanism to build resilience and non-technical skills in undergraduate nurses undertaking clinical placement. Int J Ment Health Nurs. 2020;29:1. 10.1111/inm.12607 31127972

[22] Cao Y, Gao L, Fan L, Jiao M, Li Y, Ma Y. The influence of emotional intelligence on job burnout of healthcare workers and mediating role of workplace violence: A cross sectional study. Front Public Health. 2022;10:892421. 35646806 10.3389/fpubh.2022.892421 PMC9130825

[23] Littlejohn P. The missing link: using emotional intelligence to reduce workplace stress and workplace violence in our nursing and other health care professions. J Prof Nurs. 2012;28(6):360–8. 23158199 10.1016/j.profnurs.2012.04.006

[24] Atta MHR, El-Sayed AAI, Alsenany SA, Hammad HAH, Elzohairy NW, Asal MGR. Navigating transition shock: the role of system thinking in enhancing nursing process competency among early career nurses. Worldviews evidence‐based Nurs. 2024;21(6):611–25. 10.1111/wvn.12757. 10.1111/wvn.12757 39572034

[25] Fteiha M, Awwad N. Emotional intelligence and its relationship with stress coping style. Health Psychol Open. 2020;7(2):2055102920970416. 33224513 10.1177/2055102920970416 PMC7656878

[26] Bandura A. Social foundations of thought and action: A social cognitive theory. Prentice-Hall; 1986.

[27] Alenezi A. The Impact of Resilience on Workplace Violence Experienced by Mental Health Nurses: A Cross-Sectional Survey. 2024;2024(1):4449445. 10.1155/2024/4449445 PMC11918818 40224751

[28] Xu J, Zhang L, Ji Q, Ji P, Chen Y, Song M, et al. Nursing students’ emotional empathy, emotional intelligence and higher education-related stress: a cross-sectional study. BMC Nurs. 2023;22(1):437. 37981672 10.1186/s12912-023-01607-z PMC10658862

[29] Diffley DMDM. Fostering resilience in nursing students in the academic setting: A systematic review. J Nurs Educ. 2022;1(5):229–35. 10.3928/01484834-20220303-03 35522758

[30] Karabey T, Çevik BE, Süha BK, Greco G. Determination of nursing students’ resilience levels, care behaviors, and violence management competencies: A descriptive, Cross-Sectional, and relational study. Perspect Psychiatr Care. 2023;2023(1).

[31] Aryuwat P, Asp M, Lovenmark A, Radabutr M, Holmgren J. An integrative review of resilience among nursing students in the context of nursing education. Nurs Open. 2023;10(5):2793–818. 36564896 10.1002/nop2.1559 PMC10077422

[32] Luo M, Pang J, Xie S, Xu H, Yan J. A study of the correlation between residents’ humanistic care skills and their level of emotional intelligence-A cross-sectional survey. BMC Med Educ. 2024;24(1):1136. 39402556 10.1186/s12909-024-06097-4 PMC11472458

[33] Ang WHLY. Trait emotional intelligence as a predictor of resilience among undergraduate nursing students: A structural equation modelling approach. Nurse Educ Today. 2024;1:136. 10.1016/j.nedt.2024.106132 38395026

[34] Yu J, Mei X, Zeng Y, Yuan D, Yu Y, Ye Z. Associations among emotional intelligence, resilience and humanistic caring ability in nursing postgraduates: a response surface analysis and moderated mediation model. Res Square. 2023:1–17.

[35] Aksoy FBS, Özsaban A. Nursing students’ exposure to violence in clinical practice and violence management competence levels. Nurse Educ Today. 2024;2024(1):139106237. 10.1016/j.nedt.2024.106237 38735095

[36] Huaman N, Morales-Garcia WC, Castillo-Blanco R, Saintila J, Huancahuire-Vega S, Morales-Garcia SB, et al. An explanatory model of work-family conflict and resilience as predictors of job satisfaction in nurses: the mediating role of work engagement and communication skills. J Prim Care Community Health. 2023;14:21501319231151380. 36718818 10.1177/21501319231151380 PMC9893370

[37] Martinez AJS. Enhancing nursing students’ competency skills with a workplace violence nursing simulation: translating knowledge into practice. SAGE Open Nurs. 2019;5:2377960819843696. 33415232 10.1177/2377960819843696 PMC7774437

[38] Budler LC, Gosak L, Vrbnjak D, Pajnkihar M, Štiglic G. Emotional intelligence among nursing students: findings from a longitudinal study. Healthc (Basel). 2022;10(10). 10.3390/healthcare10102032 PMC9601576 36292477

[39] El-Sayed AAI, Alsenany SA, Atta MHR, Othman AA, Asal MGR. Navigating toxicity: investigating the interplay between workplace gaslighting, workaholism, and agility among nurses. Nurs Inq. 2025;32(1):e12697. 10.1111/nin.12697. 39803721 10.1111/nin.12697

[40] Dafny HAMC, Champion S, Pearson V, Hines S, Brown S, Phillips C, Waheed N, Cabilan CJ, Johnston S. Interventions to prevent or manage workplace violence against student nurses during clinical placement: a systematic review protocol. JBI Evid Synthesis. 2024;1(5):881–8. 10.11124/JBIES-22-00441 38126266

[41] Li LLX, Ni J. A cross-sectional survey on the relationship between workplace psychological violence and empathy among Chinese nurses: the mediation role of resilience. BMC Nurs. 2024;1(1):85. 10.1186/s12912-024-01734-1 PMC10832225 38302970

